# Supplementary material for: Spatiotemporal prediction of COVID-19 cases using inter- and intra-county proxies of human interactions
Source: Nat Commun. 2021 Nov 8;12:6440. doi: 10.1038/s41467-021-26742-6 (PMC8576047; doi:10.1038/s41467-021-26742-6)
Supplement: Supplementary file 1 — Supplementary Information [file 41467_2021_26742_MOESM1_ESM.docx]

# Spatiotemporal Prediction of COVID-19 Cases using Inter- and Intra-County Proxies of Human Interactions

**Behzad Vahedi^1*^, Morteza Karimzadeh^1,^ Hamidreza Zoraghein^2^**

**^1^ Department of Geography, University of Colorado Boulder, Boulder, USA**

**^2^ Social and Behavioral Science Research, Population Council, New York, USA**

**^*^Email:** [**Behzad@colorado.edu**](mailto:Behzad@colorado.edu)

**Supplementary Information**

# Feature engineering

The base features that we used in the TXGB and all STXGB models, including socioeconomic and demographic variables, temperature variables, temporally-lagged weekly change in cumulative incidence rates, and temporally-lagged weekly average of incidence rates were generated as follows:

# Socioeconomic variables

Socioeconomic, demographic, and climatic variables are shown to be correlated with the spread of COVID-19^1,2^. To account for the effect of the socioeconomic and demographic composition of each county on the number of its new COVID-19 cases, we used a set of 10 socioeconomic variables that are summarized in Table 5 (main article). These variables were gathered from the 5-year American Community Survey (ACS) dataset collected between 2014-2018. We downloaded the data from the IPUMS National Historical GIS portal^3^.

Political affiliation has also been shown to affect an individual’s behavior in response to policies for COVID-19 preventation^4^. Therefore, we included the percentage of the population who voted for the republican party presidential candidate in the 2016 general election. Presidential election data from MIT Election Data and Science Lab^5^ were used to generate this feature.

# Temperature variables

We used a dataset of daily maximum and minimum surface temperatures of the U.S. from NOAA^6^ to generate weekly minimum and maximum temperature features calculated for inhabited areas of counties. This dataset provides the surface temperatures since as early as May 1, 2010. We separately retrieved the U.S. Populated Place Areas, including census-designated places, consolidated cities, and incorporated places to identify inhabited areas in counties. We projected and resampled daily maximum and minimum temperature surfaces from their original 0.5-degree spatial resolution to 1 KM resolution cells to ensure that there was at least one cell in each county. We then averaged values of all cells intersecting U.S. Populated Place Areas of a county to estimate its minimum and maximum temperatures. For three counties with no intersection between temperature observation cells and Populated Place Areas, we applied spatial imputation and used the average minimum and maximum temperature values of their neighboring counties. Finally, we calculated the weekly average minimum and maximum temperatures of each county as base features in all of our models.

# Weekly change in incidence rates

We defined the COVID-19 incidence rate of a county at time *t* as its number of cumulative cases per 10,000 population at *t* and used the weekly change in incidence rates, instead of weekly new cases, as a predictive feature in our models. This has two advantages: first, the incidence rates are normalized by population, and thus, provide a more realistic measure of the severity of COVID-19 in each county. Second, the distribution of county-level weekly new cases across the US is highly skewed towards populated areas and, therefore, could negatively impact the performance of machine learning algorithms.

To measure county-level incidence rates at time *t*, we used the number of cumulative confirmed COVID-19 cases at *t*, published by the Johns Hopkins University’s Center for Systems Science and Engineering (JHU CSSE). We first smoothed the number of daily confirmed cases using a seven-day rolling average to reduce the effect of inconsistencies or delays in daily testing and reporting on our training data. We then derived the number of new cases per 10,000 population in each county during a week (starting on Sunday and ending on Saturday, both inclusive). We included natural log-transformed values of change in incidence rate during four weekly temporal-lags (*t*-1,…,*t*-4), and the average of incidence rate during the fourth-lagged week (*t*-4) as features in all of our models.

# Summary of forecast dates

Supplementary Table 1 summarizes the forecast dates, one-week and 4-week ahead prediction horizons, and training data size. We evaluated STXGB models against the COVIDhub- Baseline and Ensemble models across the 14 forecast dates listed in this table by generating predictions for unseen data in 1- to 4-weeks ahead prediction horizons.

Supplementary Table 1. **Summary of forecast dates**. 1- and 4-week ahead prediction target dates, and training data used for machine learning algorithm selection, as well as comparison of STXGB-FB and STXGB-SG models with COVIDhub models

| **Forecast date** | **1-week prediction horizon target date** | **4-week prediction horizon target date** | **Training data end date** | **# training samples per county** |
| --- | --- | --- | --- | --- |
| 2020/10/25 | 2020/10/31 | 2020/11/21 | 2020/10/24 | 30 |
| 2020/11/01 | 2020/11/07 | 2020/11/28 | 2020/10/31 | 31 |
| 2020/11/08 | 2020/11/14 | 2020/12/05 | 2020/11/07 | 32 |
| 2020/11/15 | 2020/11/21 | 2020/12/12 | 2020/11/14 | 33 |
| 2020/11/22 | 2020/11/28 | 2020/12/19 | 2020/11/21 | 34 |
| 2020/11/29 | 2020/12/05 | 2020/12/26 | 2020/11/28 | 35 |
| 2020/12/06 | 2020/12/12 | 2021/01/02 | 2020/12/05 | 36 |
| 2020/12/13 | 2020/12/19 | 2021/01/09 | 2020/12/12 | 37 |
| 2020/12/20 | 2020/12/26 | 2021/01/16 | 2020/12/19 | 38 |
| 2020/12/27 | 2021/01/02 | 2021/01/23 | 2020/12/26 | 39 |
| 2021/01/03 | 2021/01/09 | 2021/01/30 | 2021/01/02 | 40 |
| 2021/01/10 | 2021/01/16 | 2021/02/06 | 2021/01/09 | 41 |
| 2021/01/17 | 2021/01/23 | 2021/02/13 | 2021/01/16 | 42 |
| 2021/01/24 | 2021/01/30 | 2021/02/20 | 2021/01/23 | 43 |

# Calculating an Urbanicity index for each county

The level of urbanicity of counties has implications on the speed of spread of the virus, albeit, urban areas are typically better equipped with health care facilities. To incorporate the urbanicity of counties in our models, we estimated an urbanization index for each county using the Socioeconomic Data and Applications Center’s (SEDAC) Global COVID-19 viewer^7^. The urbanization component of the Global COVID-19 viewer comes from the GHS Settlement Model Layers (GHS-SMOD), a product of the European Commission’s Global Human Settlement Layer (GHSL)^8,9^. To assign an urbanization index, which incorporates both urban land and population, for each county, we multiplied its number of inhabitants in classes “urban center”, “dense urban area” and “semi-dense urban area” by their corresponding class land areas, and then divided the resulting value by the multiplication of the county’s total population and land area. The index ranges from 0 to 1, and higher values indicate relatively stronger compliance to the urban lifestyle. Counties with values larger than 0.5 were considered urban-majority in this study. We used the rural/urban categorization only for investigating errors.

# Machine Learning Models: Implementation and Validation

For data processing and cleaning, we used open-source Python packages such as NumPy (version 1.20.1), Pandas (version 1.2.3), and GeoPandas (version 0.8.1). We implemented the XGB algorithm using the XGBoost^10^ software library (version 1.4.0), FFNN and LSTM algorithms using Tensorflow^11^ (version 2.4.1), and RF and SGB algorithms using Scikit-learn^12^ (version 0.24.1), all in the Python programming language. In the FFNN, we included two hidden layers with 150 nodes in each layer and used REctified Linear Unit (RELU) function to activate all layers. Each hidden layer was accompanied with a dropout layer (with a rate of 0.1). We optimized the network using Adam optimizer to minimize Mean Squared Error as the loss function. Since the network was trained for a regression task, the output layer had only one node. To develop deeper networks, we also experimented with adding more layers to the network, and more neurons in each layer, but given the relatively small size of the training dataset, this did not lead to better results.

We constructed the LSTM network by including two LSTM layers, each with 20 units. Similar to the FFNN, the LSTM network had also one output unit, but we used Huber loss as the loss function.

# Hyperparameter tuning

We tuned the hyperparameters of RF, SGB, XGB, and FFNN algorithms as summarized in Supplementary Table 2 for each prediction horizon and each forecasting date. In the case of the first three algorithms, we applied a 5-fold cross-validated randomized search over the parameter space to find the value(s) that minimized error (RMSE in our models) on the validation set (not to be confused with the held out weekly testing sets, which were subsequently used to test the model on unseen weekly data). In the case of FFNN, we deployed the hyperband^13^ algorithm for tuning the network’s hyperparameters and used Mean Squared Error (MSE) to measure error. For more information regarding the definition of each parameter or the range of acceptable values, readers can refer to the documentation of each library mentioned in the previous section.

Supplementary Table 2. List of tuned hyperparameters and the range of candidate values for each in tuning machine learning algorithms.

| **Algorithm** | **Tuned Hyperparameters** | **Hyperparameter Grid** |
| --- | --- | --- |
| **RF** | max_depth  min_samples_split  min_samples_leaf  max_features | $\left\{ k \vert k\in\{1,2,..,10\} \right\}$  $\left\{ k \vert k\in\{2,3,...,50\} \right\}$  $\left\{ k \vert k\in\{2,3,...,50\} \right\}$  $\left\{ sqrt, log2 \right\}$ |
| **SGB** | learning_rate  n_estimators  subsample  max_depth  max_features | $\left\{ 0.05k \vert k\in\left\{ 1,2,..,6 \right\} \right\}$  $\left\{ 100k \vert k\in\{1,2,...,10\} \right\}$  $\left\{ 0.05k \vert k\in\{2,3,..,18\} \right\}$  $\left\{ k \vert k\in\{1,2,..,10\} \right\}$  $\left\{ sqrt, log2 \right\}$ |
| **XGB** | learning_rate  n_estimators  gamma  subsample  max_depth | $\left\{ 0.05k \vert k\in\left\{ 1,2,..,6 \right\} \right\}$  $\left\{ 100k \vert k\in\{1,2,...,10\} \right\}$  $\left\{ k \vert k\in\{1,2,..,10\} \right\}$  $\left\{ 0.05k \vert k\in\left\{ 2,3,..,10 \right\} \right\}$  $\left\{ k \vert k\in\{1,2,..,10\} \right\}$ |
| **FFNN** | Learning rate  Number of hidden layers  number of units in each hidden layer  dropout rate for each dropout layer | $\left\{ 0.01, 0.001 \right\}$  $\left\{ k \vert k\in\{2,3,..,5\} \right\}$  $\left\{ 10k \vert k\in\{2,3,..,50\} \right\}$  $\left\{ 0.05k \vert k\in\left\{ 0,1,..,6 \right\} \right\}$ |

# Alternative model settings

To potentially improve the performance of our STXGB models, we experimented with various engineered features, but those presented in this article outperformed the alternative combinations. For instance, in addition to using the natural logarithm of the change in incidence rates (Table 5), we also used the squared value of logged change in incidence rates to potentially model the exponential increase in cases. We also experimented with new cases (instead of incidence rates), and for both settings, trained and evaluated the models with or without the logarithmic transformation of the target and lag variables. The average RMSE and MAE in all these experiments were higher than the ones resulting from using the features presented in this article. As another example of an alternative experimental setup, we used bootstrapping to sample one week's worth of additional samples from the last month of training data and added the sample to the training dataset. In effect, we increased the size of training samples by one week on each forecast date to potentially capture the latest trends of the disease spread. However, the performance of the models did not improve.

# Additional model performance reports

In this section, we present additional performance metrics of the STXGB models. Table 3 in the main article presented average prediction MAEs of the STXGB-FB and STXGB-SG models compared to the COVIDhub-Baseline model in each prediction horizon. Supplementary Figure 1 presents the RMSE values of our STXGB models in comparison with the COVIDhub-Baseline model.


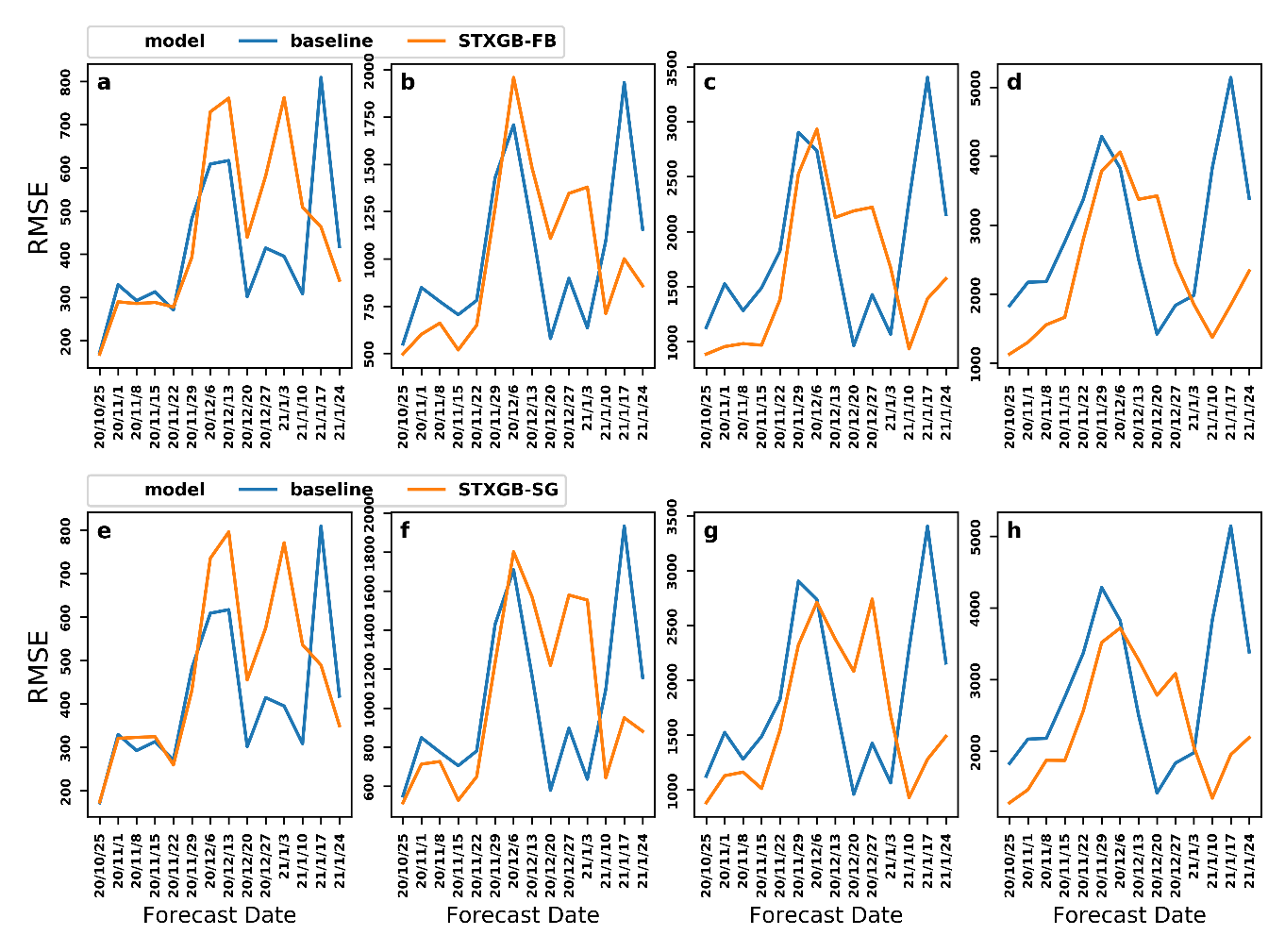


Supplementary Figure 1. **Prediction error comparison with COVIDhub-Baseline.** Prediction RMSEs of (**a-d**) STXGB-FB and (**e-h**) STXGB-SG models compared with the COVIDhub-Baseline model over four prediction horizons on 14 forecasting dates. **a** and **e**: one-week horizon, **b** and **f**: two-week horizon, **c** and **g**: three-week horizon, **d** and **h**: four-week horizon

Supplementary Table 3 presents the prediction MAEs of each model compared to the COVIDhub-Ensemble model over all forecasting dates and horizons. In the four-week prediction horizon, both STXGB-SG and STXGB-FB achieved a lower average MAE compared to the Ensemble. STXGB-SG achieved a lower average MAE in the three-week horizon as well. But the Ensemble model beats both our models in the one- and two-week prediction horizons. At the time of writing this document, this model is generated as an ensemble of 32 different models that submit weekly forecasts to the COVID-19 forecast hub and performs better than each of its constituent models. Therefore, comparing our models with the Ensemble model might be unfair as it is designed to have a better performance than the individual models.

Supplementary Table 3. **Prediction MAE in each prediction horizon compared to COVIDhub-Ensemble Mode**l. Comparison of the mean absolute prediction errors (MAE) generated by the COVID-19 Forecast Hub Ensemble model and our STXGB-FB and STXGB-SG models in 1- to 4-week prediction horizons across all forecasting dates. Values in parantheses show percentage of change compared to COVID-Hub Baseline.

| **Forecast Date** | **Model** | **1-week horizon** | | **2-week horizon** | | **3-week horizon** | | **4-week horizon** | |
| --- | --- | --- | --- | --- | --- | --- | --- | --- | --- |
| 2020/10/25 | Ensemble | 48.67 |  | 170.91 |  | 360.95 |  | 611.65 |  |
|  | STXGB-SG | 40.67 | (16.44) | 126.18 | (26.17) | 232.9 | (35.48) | 321.59 | (47.42) |
|  | STXGB-FB | 43.69 | (10.23) | 132.31 | (22.58) | 246.62 | (31.67) | 363.54 | (40.56) |
| 2020/11/01 | Ensemble | 87.39 |  | 235.17 |  | 439.86 |  | 630.69 |  |
|  | STXGB-SG | 81.72 | (6.49) | 179.49 | (23.68) | 281.52 | (36.00) | 334.26 | (47.00) |
|  | STXGB-FB | 88.54 | (-1.32) | 199.16 | (15.31) | 318.9 | (27.50) | 391.94 | (37.86) |
| 2020/11/08 | Ensemble | 99.31 |  | 244.29 |  | 378.41 |  | 580.38 |  |
|  | STXGB-SG | 98.84 | (0.47) | 213.43 | (12.63) | 307.83 | (18.65) | 445.39 | (23.26) |
|  | STXGB-FB | 109.68 | (-10.44) | 235.82 | (3.47) | 358.21 | (5.34) | 488.84 | (15.77) |
| 2020/11/15 | Ensemble | 87.09 |  | 187.29 |  | 347.55 |  | 577.36 |  |
|  | STXGB-SG | 89.74 | (-3.04) | 161.27 | (13.89) | 262.67 | (24.42) | 409.46 | (29.08) |
|  | STXGB-FB | 98.56 | (-13.17) | 159.78 | (14.69) | 266.9 | (23.21) | 427.58 | (25.94) |
| 2020/11/22 | Ensemble | 75.62 |  | 172.84 |  | 345.53 |  | 564.28 |  |
|  | STXGB-SG | 76.95 | (-1.76) | 159.06 | (7.97) | 296.2 | (14.28) | 485.05 | (14.04) |
|  | STXGB-FB | 75.67 | (-0.07) | 162.18 | (6.17) | 299.72 | (13.26) | 479.08 | (15.10) |
| 2020/11/29 | Ensemble | 101.78 |  | 251.15 |  | 432.85 |  | 596.89 |  |
|  | STXGB-SG | 95.16 | (6.50) | 228.77 | (8.91) | 411.15 | (5.01) | 644.72 | (-8.01) |
|  | STXGB-FB | 101.43 | (0.34) | 235.5 | (6.23) | 410.3 | (5.21) | 610.65 | (-2.31) |
| 2020/12/06 | Ensemble | 103.95 |  | 225.17 |  | 339.76 |  | 471.25 |  |
|  | STXGB-SG | 118.13 | (-13.64) | 268.89 | (-19.42) | 422.21 | (-24.27) | 579.09 | (-22.88) |
|  | STXGB-FB | 121.03 | (-16.43) | 263.36 | (-16.96) | 403.66 | (-18.81) | 553.17 | (-17.38) |
| 2020/12/03 | Ensemble | 88.73 |  | 181.11 |  | 278.63 |  | 386.31 |  |
|  | STXGB-SG | 124.1 | (-39.86) | 264.98 | (-46.31) | 369.69 | (-32.68) | 506.93 | (-31.22) |
|  | STXGB-FB | 130.57 | (-47.15) | 269.47 | (-48.79) | 375.31 | (-34.70) | 473.47 | (-22.56) |
| 2020/12/20 | Ensemble | 66.82 |  | 127.55 |  | 218.71 |  | 324.64 |  |
|  | STXGB-SG | 103.35 | (-54.67) | 193.56 | (-51.75) | 334.42 | (-52.91) | 472.4 | (-45.52) |
|  | STXGB-FB | 106.6 | (-59.53) | 195.59 | (-53.34) | 270.53 | (-23.69) | 372.13 | (-14.63) |
| 2020/12/27 | Ensemble | 86.94 |  | 239.51 |  | 378.2 |  | 470.39 |  |
|  | STXGB-SG | 88.44 | (-1.73) | 204.95 | (14.43) | 317.28 | (16.11) | 371.4 | (21.04) |
|  | STXGB-FB | 84.93 | (2.31) | 213.04 | (11.05) | 321.21 | (15.07) | 379.03 | (19.42) |
| 2021/01/03 | Ensemble | 130.94 |  | 228.57 |  | 274.22 |  | 316.1 |  |
|  | STXGB-SG | 157.44 | (-20.24) | 234.94 | (-2.79) | 281.92 | (-2.81) | 340.38 | (-7.68) |
|  | STXGB-FB | 158.01 | (-20.67) | 233.99 | (-2.37) | 266.22 | (2.92) | 340.68 | (-7.78) |
| 2021/01/10 | Ensemble | 73.24 |  | 186.69 |  | 348.53 |  | 571.11 |  |
|  | STXGB-SG | 86.8 | (-18.51) | 173.5 | (7.07) | 261.43 | (24.99) | 375.61 | (34.23) |
|  | STXGB-FB | 85.7 | (-17.01) | 179.45 | (3.88) | 276.95 | (20.54) | 382.04 | (33.11) |
| 2021/01/17 | Ensemble | 89.94 |  | 203.81 |  | 383.2 |  | 600.86 |  |
|  | STXGB-SG | 124.89 | (-38.86) | 242.32 | (-18.90) | 360.33 | (5.97) | 494.5 | (17.70) |
|  | STXGB-FB | 134.43 | (-49.47) | 256.55 | (-25.88) | 357.83 | (6.62) | 533.68 | (11.18) |
| 2021/01/24 | Ensemble | 52.17 |  | 126.75 |  | 226.5 |  | 354.64 |  |
|  | STXGB-SG | 86.29 | (-65.40) | 208.64 | (-64.61) | 383.57 | (-69.35) | 547.35 | (-54.34) |
|  | STXGB-FB | 77.83 | (-49.19) | 192.32 | (-51.73) | 343.42 | (-51.62) | 519.37 | (-46.45) |
| **Average Pct. Change** | **STXGB-SG** | **-16.27** | | **-6.36** | | **-0.08** | | **4.58** | |
|  | **STXGB-FB** | **-19.40** | | **-8.26** | | **1.61** | | **6.27** | |

Supplementary Figure 2 and Supplementary Figure 3 present the 95% Prediction Intervals (PIs) of the Ensemble model in comparison with STXGB-FB and STXGB-SG respectively, when predicting the total number of cumulative new cases over the four prediction horizons. The PIs of STXGB models are generated using the method outlined in the Methods Section of the main article. In the case of the Ensemble model, we have used the quantile predictions at the 0.025 and 0.975 levels (reported by the COVID-19 Forecast Hub) as the lower and upper interval predictions respectively, and generated the PI using a similar method to what is described for the COVID-Hub Baseline model in the main article.

The Ensemble model has wider prediction intervals compared to both STXGB models in all prediction horizons, pointing to higher overall uncertainty. All three models cover all the observed values over all forecasting dates and prediction horizons. Supplementary Figure 2 also shows that when predicting the total (national) number of new cases, the STXGB-FB generates more accurate predictions compared to the Ensemble on 4, 8, 9, and 8 forecasting dates out of the total 14 forecasting dates over one- to four-week prediction horizons respectively. STXGB-SG also achieves higher accuracies compared to the Ensemble on 3, 8, 10, and 8 forecasting dates out of the 14 forecasting dates over one- to four-week horizons respectively (Supplementary Figure 3).

| 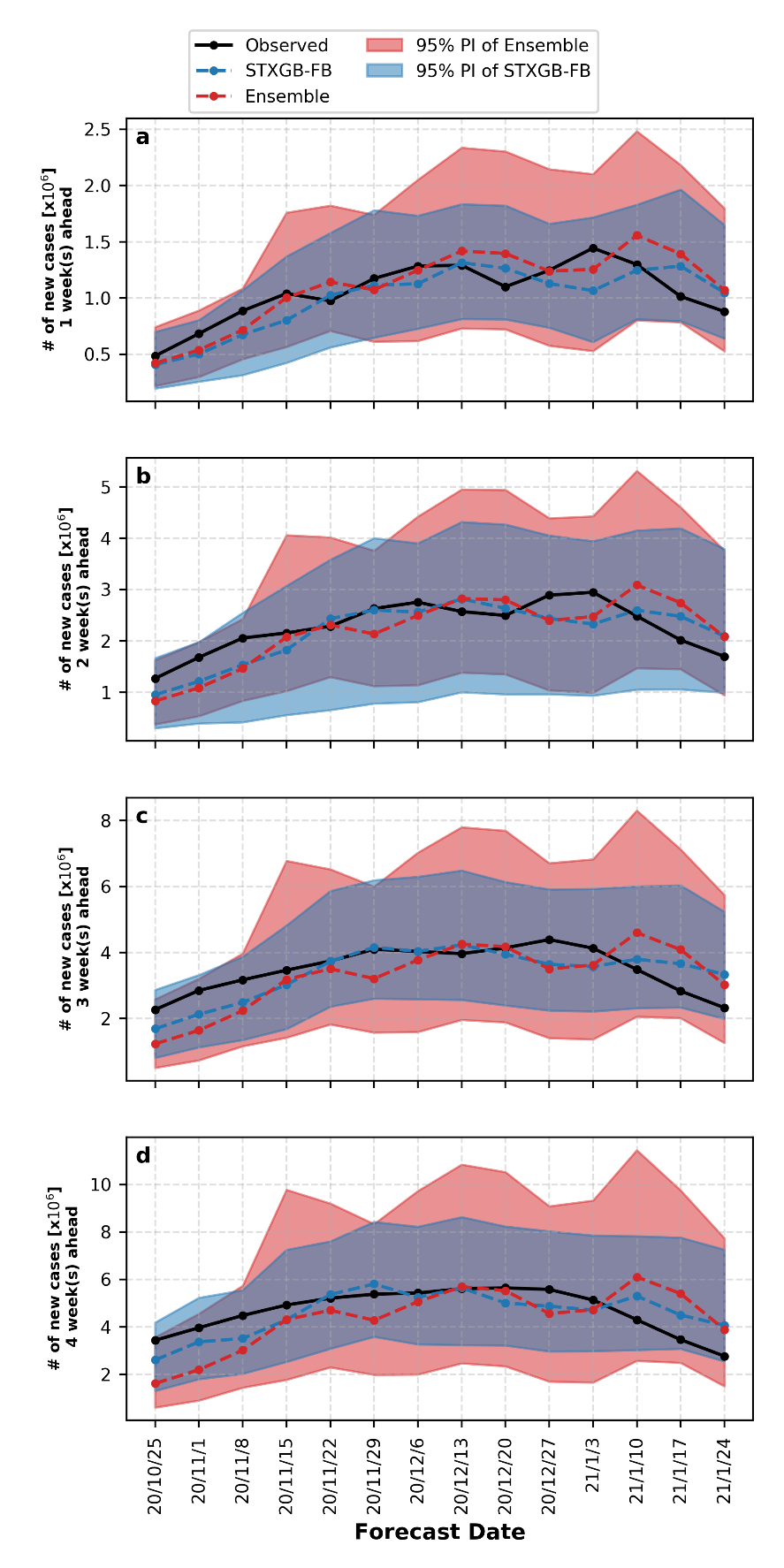  Supplementary Figure 2. **95% prediction intervals.** 95% prediction interval of the STXGB-FB model compared to the COVIDhub-Ensemble over four prediction horizons. **a** one-week horizon, **b** two-week horizon, **c** three-week horizon, and **d** four-week horizon. |
| --- |
| 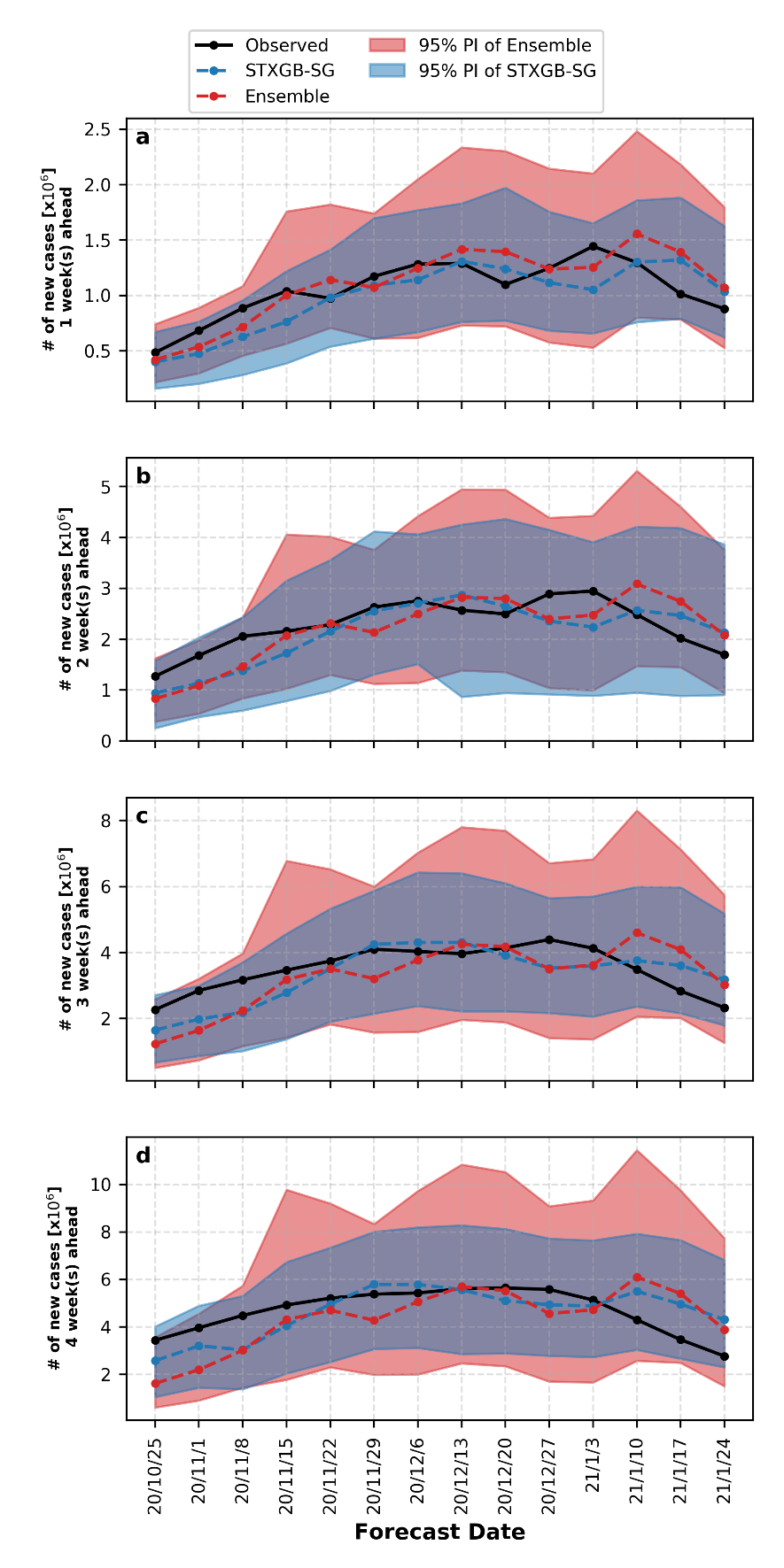  Supplementary Figure 3. **95% prediction intervals.** 95% prediction interval of the STXGB-SG model compared to the COVIDhub-Ensemble over four prediction horizons. **a** one-week horizon, **b** two-week horizon, **c** three-week horizon, and **d** four-week horizon. |

In the “Spatial distribution of errors” subsection of the Results section of the article, we described the differences between STXGB-FB and the COVIDhub-Baseline model when predicting new csaes in rural and urban counties. Fig. 6 (e-h) in the main article presented the comparison between the two models when predicting number of cases per 10k population and the Supplementary Fig. 4 presents the same comparison, but when predicting number of cases (not normalized by population). Please refer to the Results section of the article for a detailed comparison.


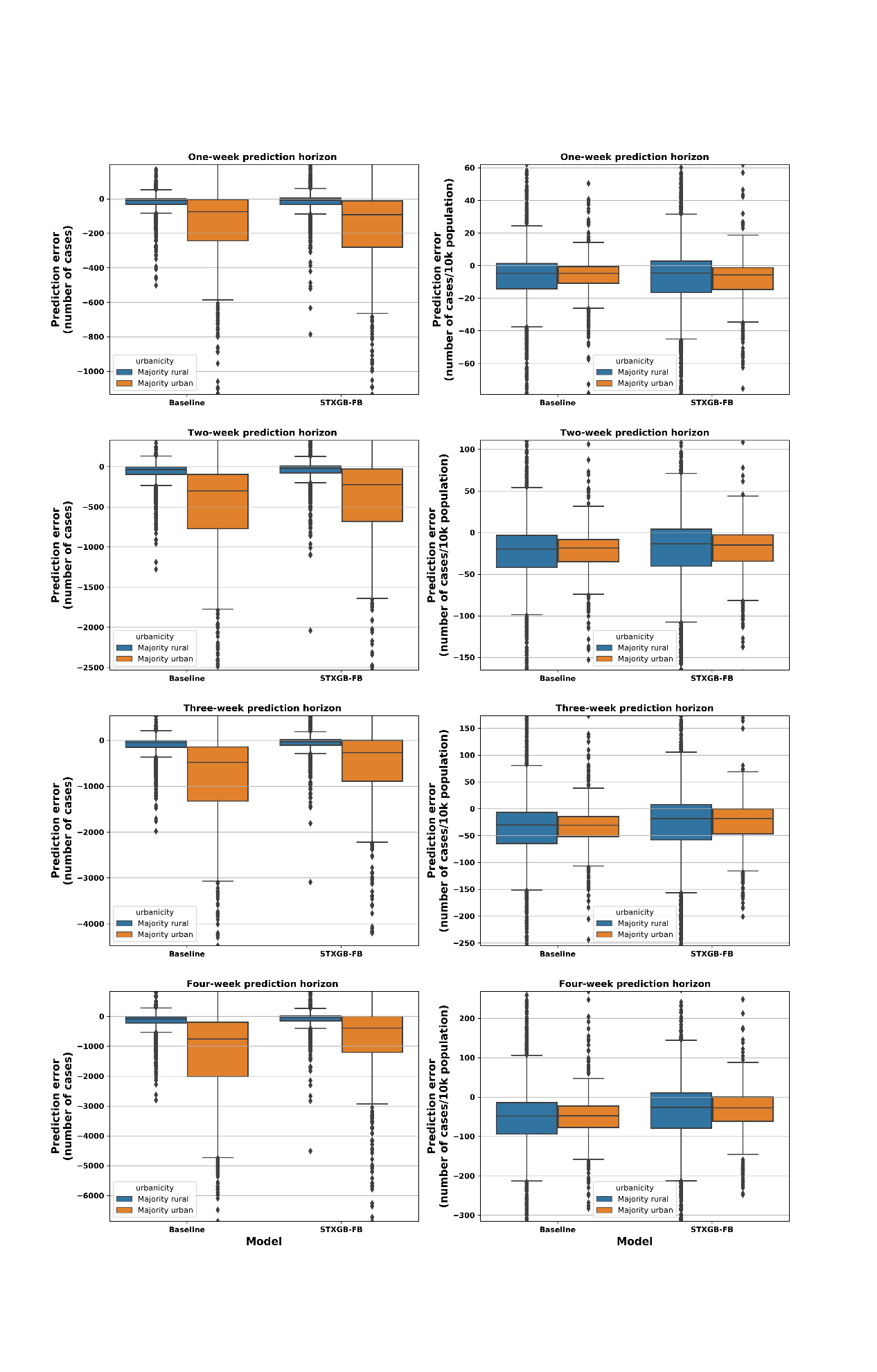


Supplementary Figure 4. Prediction errors of the number of new cases in rural and urban counties on the Nov. 8 forecast date across four prediction horizons. The higher and lower 1% of counties are trimmed from the plot view. Data are presented as means ± SEM (n=2391 for majority rural group and n=712 for majority urban group). Whiskers represent 1.5 IQR (interquartile range).

# Model Interpretability

To investigate the differences in the performance of the STXGB-FB model compared to the STXGB-SG on average, i.e. whether social media-derived features have higher predictive power compared to the cell-phone-derived features, we analyzed the “feature importance” of both models over all forecasting dates and prediction horizons. As an example, Supplementary Figure 5 presents the importance of the features used in the STXG-FB model for the four-week prediction horizon on Nov. 8 forecasting date (the same forecasting date for which Fig. 5 e-h in the main article is generated) and Supplementary Figure 6 presents the importance of features used in STXG-SG, for the same forecasting date and prediction horizon. The importance of a features is measured by the amount of change (decrease) in variance brought by a specific feature, to the tree branch where that feature is used for splitting^[[1]](#footnote-1)^. The higher the importance of a feature, the higher is the reduction in variance. It is important to note that this criterion provides a relative measure to compare the importance of different features and the absolute values are not meant to be interpreted as the actual importance of any specific feature.

As seen in Supplementary Figure 5 and 6, one-week lagged change in incidence rate (Ln (Δ cumulative incidence rate +1)_t-1_) is the most important feature in both models by a considerable margin. This means that the change in the incidence rate of COVID-19 during the most recent week is the most important predictor of the change in the immediate next week.

In STXGB-FB, one-week lagged change in SPC (Δ SPC _t-1_ ) is consistently the second most important feature, over all forecasting dates and prediction horizons (56 out of 56 predictions), and other movement-related features usually achieve the next highest importance scores. However, in the case of STXGB-SG, one-week lagged change in FPC (Δ FPC _t-1_), which is the direct counterpart for Δ SPC _t-1_, does not achieve the same level of relative importance; it is the second most important feature in only 11 out of 56 predictions (over 14 forecasting dates and 4 prediction horizons) and its relative importance decreases as the prediction horizon increases. Other features used in STXGB-SG, such as mean (FPC)_t-4_ and percentage change in completely_home_device_count (the number of devices that did not leave their home location during a day) at different temporal lags sporadically achieve high importance which results in a less stable list of most important features compared to STXGB-FB. This could partially explain the difference in the performance of the two models. Furthermore, this comparison shows that SPC is generally a more predictive feature compared to FPC. Please refer to Table 5 and Methods Section of the main article for a more detailed explanation of each feature.

| 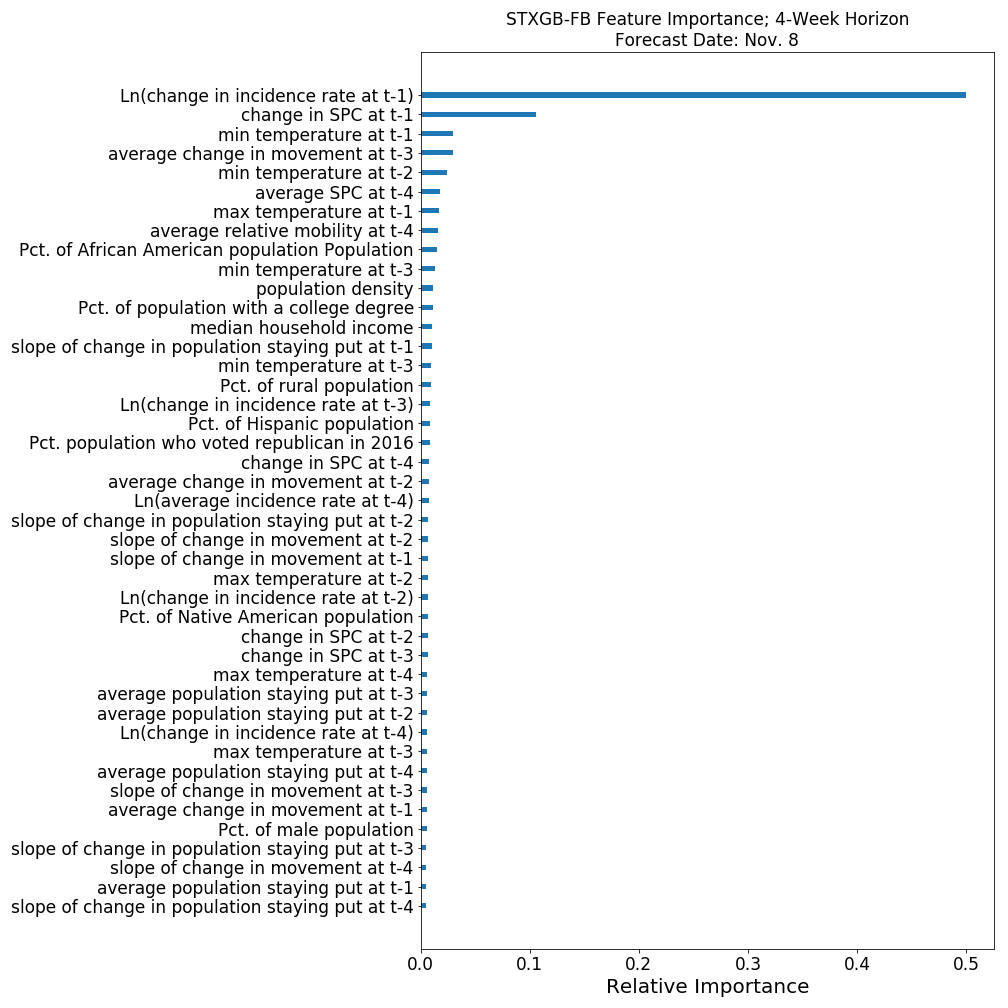  Supplementary Figure 5. **Importance of features used in the STXGB-FB model on Nov. 8 forecasting dates for four-week prediction.** This plot shows the relative importance of features as measured by the reduction in variance they provide. Δ SPC t-1 is the second most important feature in this, and all other, forecasting dates/prediction horizons. |
| --- |

| 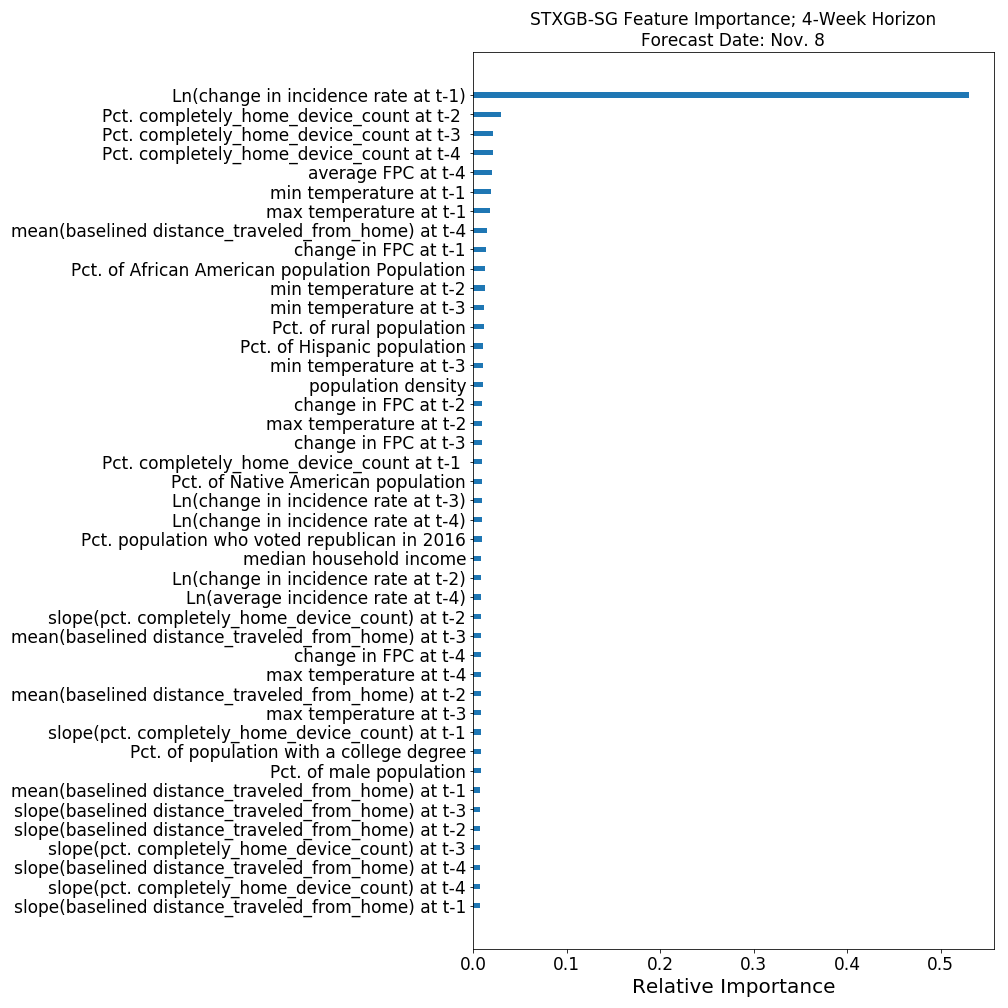  Supplementary Figure 6. **Importance of features used in the STXGB-SG model on Nov. 8 forecasting dates for four-week prediction.** This plot shows the relative importance of features as measured by the reduction in variance they provide. Please note that Δ FPC t-1 is the ninth most important feature at this forecasting date/prediction horizon. |
| --- |

# Model comparison in counties with the highest number of new cases

In addition to the comparisons presented in the main article (for the coterminous US), we compared the performance of our STXGB models with the COVID-Hub Baseline model in 50 counties with the highest numbers of weekly new COVID-19 cases. To do so, on each forecasting date, we selected 50 counties (1.6% of counties in the coterminous US) with the highest number of new cases in the one-week ahead horizon, and calculated prediction MAEs of all three models aggregated over those counties. Supplementary Table 4 presents the prediction MAEs of the COVID-Hub Baseline, STXGB-FB, and STXGB-SG models for the top 50 counties at each forecasting date across the four prediction horizons. Comparing this table with Table 3 (main article) shows that all three models perform considerably worse in these counties compared to all of the coterminous US. This is expected as these are the counties that have experienced either the highest increase in the number of weekly new cases, or are reporting (with latency) cases that (should) have been observed in the previous weeks, but went underreported. Predicting such an increase is challenging for models that are calibrated on the national level and assume consistent reporting. Regardless, STXGB-FB and STXGB-SG perform better than the COVID-Hub Baseline in two-, three-, and four-week ahead prediction horizons, but do not perform as well in the one-week prediction horizon.

Supplementary Table 4. **Prediction** **MAE in Prediction Horizon.** Comparison of the mean absolute prediction errors (MAE) generated by the COVID-Hub Baseline model and our STXGB-FB and STXGB-SG models for 50 counties with the highest number of new cases in one week ahead of each forecast date over 1- to 4-week prediction horizons. Values in parantheses show percentage of change compared to COVID-Hub Baseline.

| **Forecast Date** | **Model** | **1-week horizon** | | **2-week horizon** | | **3-week horizon** | | **4-week horizon** | |
| --- | --- | --- | --- | --- | --- | --- | --- | --- | --- |
| 10/25/20 | Baseline | 584.2 |  | 2206.4 | | 5135.9 | | 9172.8 | |
|  | STXGB-FB | 691.7 | (-18.4) | 2087.4 | (5.4) | 3978.8 | (22.5) | 5980.4 | (34.8) |
|  | STXGB-SG | 743.1 | (-27.2) | 2244.7 | (-1.7) | 4307.9 | (16.1) | 6867.1 | (25.1) |
| 11/1/20 | Baseline | 1057.5 | | 3724.8 | | 7457.3 | | 10859.9 | |
|  | STXGB-FB | 1226.2 | (-16.0) | 2978.2 | (20.0) | 4877.9 | (34.6) | 6293.8 | (42.0) |
|  | STXGB-SG | 1399.4 | (-32.3) | 3622.1 | (2.8) | 5914.8 | (20.7) | 6446.6 | (40.6) |
| 11/8/20 | Baseline | 1117.3 | | 3668.7 | | 5949.9 | | 9557.3 | |
|  | STXGB-FB | 1425.5 | (-27.6) | 3577.6 | (2.5) | 4645.7 | (21.9) | 6717.9 | (29.7) |
|  | STXGB-SG | 1588.0 | (-42.1) | 4102.6 | (-11.8) | 6055.5 | (-1.8) | 9034.1 | (5.5) |
| 11/15/20 | Baseline | 1195.5 | | 2808.8 | | 5753.5 | | 10348.4 | |
|  | STXGB-FB | 1524.7 | (-27.5) | 2320.8 | (17.4) | 3957.1 | (31.2) | 7481.4 | (27.7) |
|  | STXGB-SG | 1670.5 | (-39.7) | 2597.8 | (7.5) | 4547.9 | (21.0) | 7509.9 | (27.4) |
| 11/22/20 | Baseline | 1378.3 | | 3056.9 | | 6895.9 | | 12201.9 | |
|  | STXGB-FB | 1116.5 | (19.0) | 3026.0 | (1.0) | 6248.8 | (9.4) | 10034.7 | (17.8) |
|  | STXGB-SG | 1034.1 | (25.0) | 2771.5 | (9.3) | 6046.2 | (12.3) | 11845.9 | (2.9) |
| 11/29/20 | Baseline | 1359.9 | | 4605.8 | | 9390.2 | | 13759.5 | |
|  | STXGB-FB | 1566.6 | (-15.2) | 4796.3 | (-4.1) | 8887.2 | (5.4) | 13900.0 | (-1.0) |
|  | STXGB-SG | 1583.8 | (-16.5) | 4831.8 | (-4.9) | 10034.7 | (-6.9) | 14730.4 | (-7.1) |
| 12/6/20 | Baseline | 1958.2 | | 5287.0 | | 8806.9 | | 12241.7 | |
|  | STXGB-FB | 2442.1 | (-24.7) | 6120.9 | (-15.8) | 9318.0 | (-5.8) | 12502.0 | (-2.1) |
|  | STXGB-SG | 2439.8 | (-24.6) | 6038.0 | (-14.2) | 9620.4 | (-9.2) | 13549.8 | (-10.7) |
| 12/13/20 | Baseline | 1894.6 | | 4197.1 | | 6805.5 | | 10121.4 | |
|  | STXGB-FB | 2439.8 | (-28.8) | 5049.7 | (-20.3) | 8227.3 | (-20.9) | 10649.9 | (-5.2) |
|  | STXGB-SG | 2439.3 | (-28.7) | 5293.4 | (-26.1) | 8245.9 | (-21.2) | 12064.7 | (-19.2) |
| 12/20/20 | Baseline | 2410.8 | | 3054.7 | | 4306.5 | | 6713.3 | |
|  | STXGB-FB | 1529.8 | (36.5) | 3973.4 | (-30.1) | 8082.1 | (-87.7) | 12859.1 | (-91.5) |
|  | STXGB-SG | 1530.1 | (36.5) | 3594.4 | (-17.7) | 7549.8 | (-75.3) | 13445.5 | (-100.3) |
| 12/27/20 | Baseline | 1615.7 | | 3731.7 | | 6125.9 | | 8661.5 | |
|  | STXGB-FB | 1894.0 | (-17.2) | 4517.4 | (-21.1) | 9682.8 | (-58.1) | 12087.7 | (-39.6) |
|  | STXGB-SG | 1951.7 | (-20.8) | 4732.8 | (-26.8) | 10611.0 | (-73.2) | 11655.0 | (-34.6) |
| 1/3/21 | Baseline | 1243.0 | | 1818.9 | | 3261.0 | | 6101.0 | |
|  | STXGB-FB | 1781.2 | (-43.3) | 2876.6 | (-58.1) | 4531.2 | (-39.0) | 9599.3 | (-57.3) |
|  | STXGB-SG | 1633.4 | (-31.4) | 2941.1 | (-61.7) | 4747.7 | (-45.6) | 6341.8 | (-3.9) |
| 1/10/21 | Baseline | 2217.9 | | 5501.7 | | 10024.6 | | 16138.1 | |
|  | STXGB-FB | 1768.4 | (20.3) | 3127.9 | (43.1) | 4354.1 | (56.6) | 7336.6 | (54.5) |
|  | STXGB-SG | 1475.4 | (33.5) | 2977.3 | (45.9) | 4455.0 | (55.6) | 7556.2 | (53.2) |
| 1/17/21 | Baseline | 4119.5 | | 8516.8 | | 14577.5 | | 21843.9 | |
|  | STXGB-FB | 1872.9 | (54.5) | 2844.3 | (66.6) | 4936.8 | (66.1) | 5018.2 | (77.0) |
|  | STXGB-SG | 2076.3 | (49.6) | 3087.9 | (63.7) | 4593.0 | (68.5) | 9452.0 | (56.7) |
| 1/24/21 | Baseline | 2444.2 | | 5465.9 | | 9688.0 | | 15326.8 | |
|  | STXGB-FB | 1168.0 | (52.2) | 2695.3 | (50.7) | 6354.0 | (34.4) | 8089.3 | (47.2) |
|  | STXGB-SG | 1288.2 | (47.3) | 3141.0 | (42.5) | 5367.8 | (44.6) | 10298.7 | (32.8) |
| **Avg. Pct. Change** | **STXGB-FB** | **-2.58** | | **4.09** | | **5.05** | | **9.57** | |
|  | **STXGB-SG** | **-5.11** | | **0.48** | | **0.40** | | **4.90** | |

To compare the uncertainty of the models in the 50 counties with the highest number of new cases, we generated 95% prediction intervals using a method similar to what is described in the main article but aggregated the lower and upper interval predictions only over those 50 counties. Supplementary Figure 7 and Supplementary Figure 8 respectively present the PIs of STXGB-FB and STXGB-SG compared to the COVID-Hub Baseline. STXGB-FB performs better than the other models and its PI covers all of the observed values, i.e. total number of new cases over the 50 counties, on each forecasting date and across all prediction horizons. The STXGB-SG slightly outperforms the COVIDhub-Baseline in this respect and its PI covers 12, 14, 13, and 14 observed values (out of a total of 14) on one- to four-week prediction horizons, while the corresponding numbers for the COVID-Hub Baseline model are 13, 11, 10, and 9.

It is important to note that the general trend of the observed numbers of new cases in the top 50 counties are similar to that of the entire coterminous US (Fig. 2i in the main article), which means that the national trend in the US is dominated by a small percentage of counties reporting very high numbers of new cases.

| 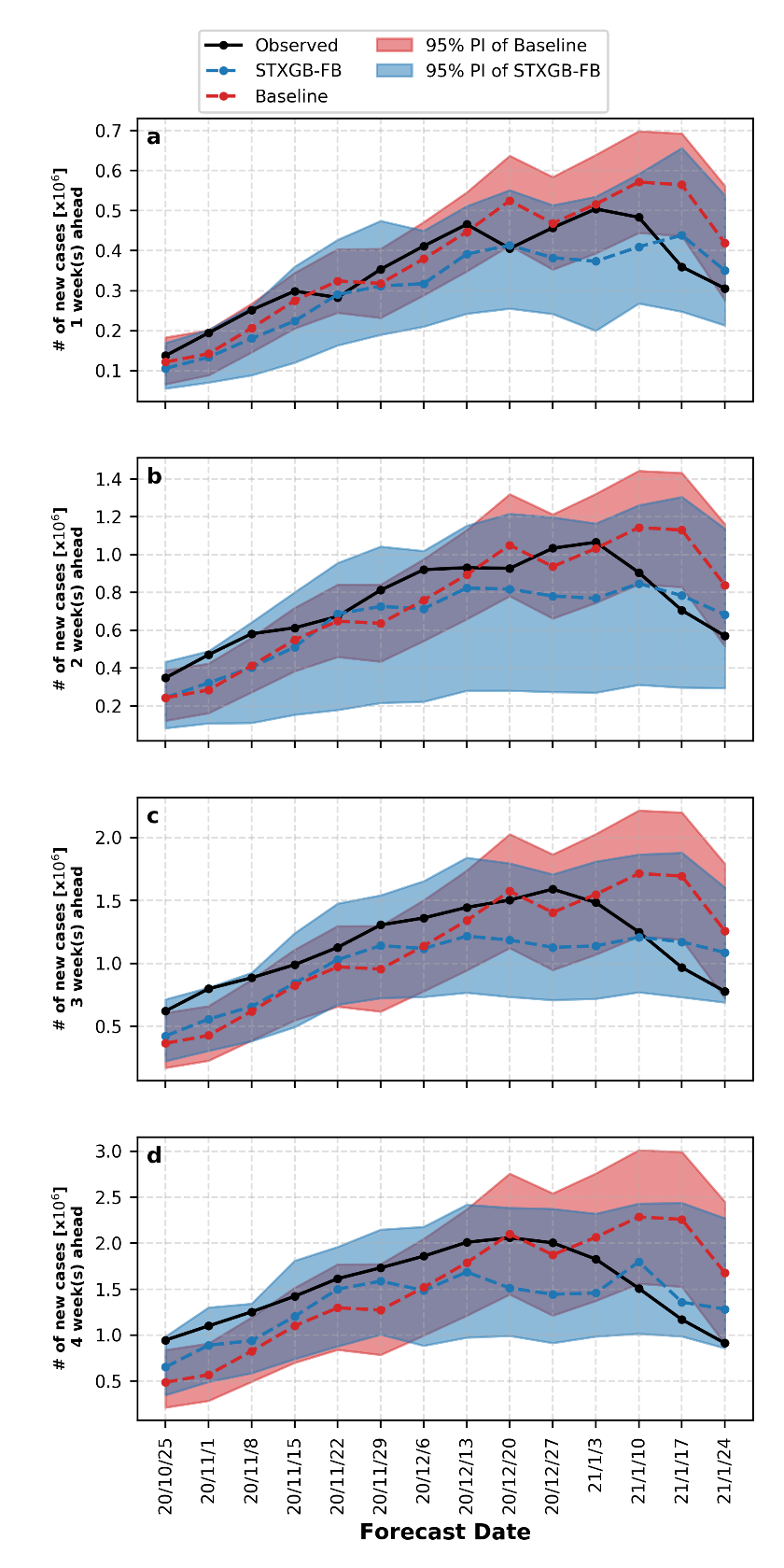  Supplementary Figure 7. **95% prediction intervals** **in 50 counties with the highest number of new cases.** 95% prediction interval of the STXGB-FB model compared to the COVID-Hub Baseline for 50 counties with the highest number of new cases in one week ahead of each forecast date over four prediction horizons. **a** one-week horizon, **b** two-week horizon, **c** three-week horizon, and **d** four-week horizon. |
| --- |
| 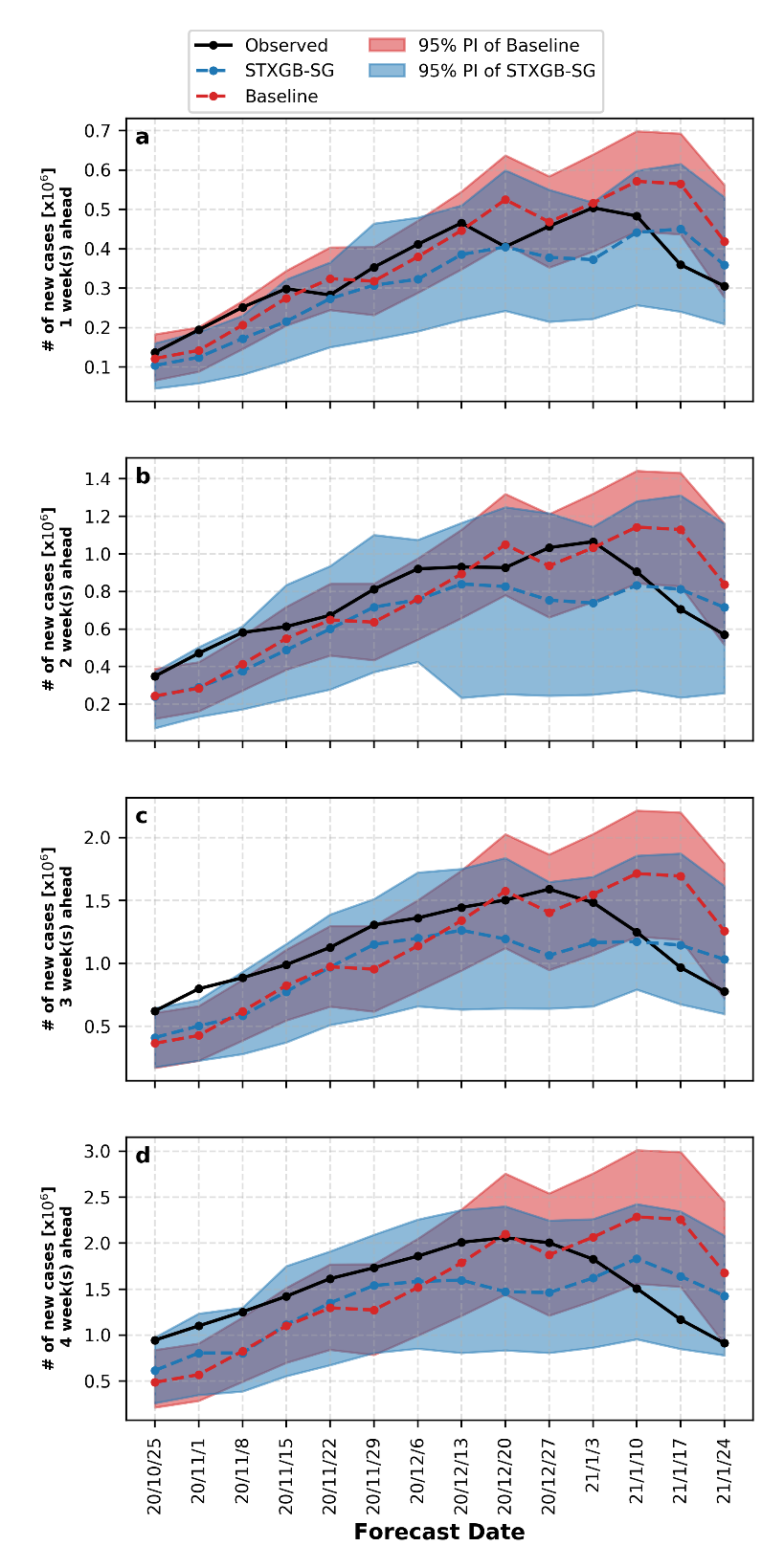  Supplementary Figure 8. **95% prediction intervals** **in 50 counties with the highest number of new cases.** 95% prediction interval of the STXGB-SG model compared to the COVID-Hub Baseline for 50 counties with the highest number of new cases in one week ahead of each forecast date over four prediction horizons. **a** one-week horizon, **b** two-week horizon, **c** three-week horizon, and **d** four-week horizon. |

**Supplementary References**

1. Bashir, M. F. *et al.* Correlation between climate indicators and COVID-19 pandemic in New York, USA. *Sci. Total Environ.* **728**, 138835 (2020).

2. Mollalo, A., Vahedi, B. & Rivera, K. M. GIS-based spatial modeling of COVID-19 incidence rate in the continental United States. *Sci. Total Environ.* **728**, 138884 (2020).

3. Manson, S., Schroeder, J., Van Riper, D., Ruggles, S., & others. IPUMS national historical geographic information system: Version 12.0 [database]. *Minneap. Univ. Minn.* **39**, (2017).

4. Grossman, G., Kim, S., Rexer, J. M. & Thirumurthy, H. Political partisanship influences behavioral responses to governors’ recommendations for COVID-19 prevention in the United States. *Proc. Natl. Acad. Sci.* **117**, 24144–24153 (2020).

5. MIT Election Data and Science Lab. *County Presidential Election Returns 2000-2016*. https://doi.org/10.7910/DVN/VOQCHQ (2018).

6. Climate Prediction Center - GIS data (Shapefile and Raster). https://www.cpc.ncep.noaa.gov/products/GIS/GIS_DATA/ (Retrieved on January 30, 2020).

7. SEDAC Global COVID-19 Viewer: Population Estimates by Age Group and Sex. https://sedac.ciesin.columbia.edu/mapping/popest/covid-19/ (Retrieved on January 30, 2020).

8. Florczyk, A. J. *et al.* GHSL data package 2019. *Luxemb. EUR* **29788**, 290498 (2019).

9. Corbane, C. *et al.* Automated global delineation of human settlements from 40 years of Landsat satellite data archives. *Big Earth Data* **3**, 140–169 (2019).

10. Chen, T. & Guestrin, C. XGBoost: A Scalable Tree Boosting System. in *Proceedings of the 22nd ACM SIGKDD International Conference on Knowledge Discovery and Data Mining* 785–794 (Association for Computing Machinery, 2016). doi:10.1145/2939672.2939785.

11. Martín Abadi *et al.* *TensorFlow: Large-Scale Machine Learning on Heterogeneous Systems*. Preprint at https://arxiv.org/abs/1603.04467 (2015).

12. Pedregosa, F. *et al.* Scikit-learn: Machine learning in Python. *J. Mach. Learn. Res.* **12**, 2825–2830 (2011).

13. Li, L., Jamieson, K., DeSalvo, G., Rostamizadeh, A. & Talwalkar, A. Hyperband: A Novel Bandit-Based Approach to Hyperparameter Optimization. *J. Mach. Learn. Res.* **18**, (2017)

1. In XGBoost library (which we used to implement STXGB models) this criterion is called gain [↑](#footnote-ref-1)
