# Supplementary material for: Spatiotemporal prediction of COVID-19 cases using inter- and intra-county proxies of human interactions
Source: Nat Commun. 2021 Nov 8;12:6440. doi: 10.1038/s41467-021-26742-6 (PMC8576047; doi:10.1038/s41467-021-26742-6)
Supplement: Supplementary file 3 — Reporting Summary [file 41467_2021_26742_MOESM3_ESM.pdf]

nature portfolio

Corresponding author(s): Behzad Vahedi

Last updated by author(s): Sep 30, 2021

Reporting Summary

Nature Portfolio wishes to improve the reproducibility of the work that we publish. This form provides structure for consistency and transparency in reporting. For further information on Nature Portfolio policies, see our Editorial Policies and the Editorial Policy Checklist.

Statistics

For all statistical analyses, confirm that the following items are present in the figure legend, table legend, main text, or Methods section.

n/a	Confirmed	
☐	☒	The exact sample size (n) for each experimental group/condition, given as a discrete number and unit of measurement
☒	☐	A statement on whether measurements were taken from distinct samples or whether the same sample was measured repeatedly
☒	☐	The statistical test(s) used AND whether they are one- or two-sided<br>*Only common tests should be described solely by name; describe more complex techniques in the Methods section.*
☒	☐	A description of all covariates tested
☒	☐	A description of any assumptions or corrections, such as tests of normality and adjustment for multiple comparisons
☐	☒	A full description of the statistical parameters including central tendency (e.g. means) or other basic estimates (e.g. regression coefficient) AND variation (e.g. standard deviation) or associated estimates of uncertainty (e.g. confidence intervals)
☒	☐	For null hypothesis testing, the test statistic (e.g. F, t, r) with confidence intervals, effect sizes, degrees of freedom and P value noted<br>*Give P values as exact values whenever suitable.*
☒	☐	For Bayesian analysis, information on the choice of priors and Markov chain Monte Carlo settings
☒	☐	For hierarchical and complex designs, identification of the appropriate level for tests and full reporting of outcomes
☒	☐	Estimates of effect sizes (e.g. Cohen's d, Pearson's r), indicating how they were calculated

Our web collection on statistics for biologists contains articles on many of the points above.

Software and code

Policy information about availability of computer code

Data collection	We did not directly collect data and thus did not use any software for data collection. All the datasets used in our analyses are publicly available for download as described in the data availability statement. The code that we used for data cleaning and preprocessing (along with the code for implementing STXGB model) is publicly available at https://github.com/geohai/COVID19-STGXB
Data analysis	For data processing and cleaning, we used open source Python packages such as numpy (version 1.20.1), pandas (version 1.2.3) and GeoPandas (version 0.8.1).<br>We implemented the STXGB algorithm using the XGBoost software library (version 1.4.0), FFNN and LSTM algorithms using Tensorflow (version 2.4.1), and RF and SGB algorithms using Scikit-learn (version 0.24.1), all in the Python programming language.

For manuscripts utilizing custom algorithms or software that are central to the research but not yet described in published literature, software must be made available to editors and reviewers. We strongly encourage code deposition in a community repository (e.g. GitHub). See the Nature Portfolio guidelines for submitting code & software for further information.

Data

Policy information about availability of data

All manuscripts must include a data availability statement. This statement should provide the following information, where applicable:
- Accession codes, unique identifiers, or web links for publicly available datasets
- A description of any restrictions on data availability
- For clinical datasets or third party data, please ensure that the statement adheres to our policy

All of the datasets used in this study are publicly available (at the time of writing this manuscript). Here we provide a brief description of each dataset. For more information, please refer to the article and supplementary information document.

We created socioeconomic features from the 5-year survey data, between 2014-2018, provided by the American Community Survey (ACS) and available at IPUMS National Historical GIS portal (https://www.nhgis.org/). The ACS publishes socioeconomic data in the US as yearly and 5-year survey data. "The 5-year estimates from ACS are period estimates that represent data collected over a period of time" (https://www.census.gov/data/developers/data-sets/acs-5year.html). "IPUMS integrates and documents survey data from around the world" (https://www.ipums.org/mission-purpose).

We used a dataset of daily maximum and minimum surface temperatures of the U.S. published by the National Oceanic and Atmospheric Administration (NOAA) to calculate weekly average minimum and maximum temperature features for inhabited areas of counties. This dataset provides the surface temperatures since as early as May 1, 2010 and is publicly available at https://ftp.cpc.ncep.noaa.gov/GIS/GRADS_GIS/GeoTIFF/TEMP/.

We used the cumulative confirmed COVID-19 cases published by the Johns Hopkins University Center for Systems Science and Engineering (JHU CSSE) to generate COVID-related features (such as number of weekly new cases, incidence rate, etc). This dataset is publicly available at https://github.com/CSSEGISandData/COVID-19/tree/master/csse_covid_19_data/csse_covid_19_time_series

Facebook's Social Connectedness Index (SCI) database is available at https://dataforgood.fb.com/tools/social-connectedness-index/ and the movement range dataset can be found at https://data.humdata.org/dataset/movement-range-maps. Both of these datasets are generated by Facebook from mobile devices that carry the Facebook application and are anonymized to protect the privacy of the users. We used these datasets to generate Facebook-related features (such as movement and social proximity to cases).

Finally, to generate SafeGraph-related features (such as movement and flow proximity to cases) we used Social Distancing Metrics dataset that is "generated using a panel of GPS pings from anonymous mobile devices". the instructions for accessing SafeGraph's Social Distancing Metrics dataset is available at https://docs.safegraph.com/docs/social-distancing-metrics.

Field-specific reporting

Please select the one below that is the best fit for your research. If you are not sure, read the appropriate sections before making your selection.

☐ Life sciences ☒ Behavioural & social sciences ☐ Ecological, evolutionary & environmental sciences

For a reference copy of the document with all sections, see nature.com/documents/nr-reporting-summary-flat.pdf

Behavioural & social sciences study design

All studies must disclose on these points even when the disclosure is negative.

Study description	In this study, we developed a spatiotemporal autoregressive model to predict county-level new cases of COVID-19 in the coterminous US using spatiotemporal lags of infection rates, human interactions, human mobility, and socioeconomic composition of counties as predictive features. All data (features) used in this study are quantitative and aggregated to the county level.
Research sample	The US and its territories has 3243 counties in total. In this study we performed our analyses on the counties in the coterminous United States (n=3103) because: 1) it is a representative sample of the entire US, and 2) not all of the datasets we used as source data were available for counties outside of the coterminous US.<br>The data we used is aggregated to the county level and is not age, gender, or race specific.
Sampling strategy	Our sampling strategy was a spatial one, meaning that we used a spatial filter to identify the counties that are within the coterminous United States. Once these counties were identified (3103 out of a total 3243 counties), we performed our analyses on all counties within this sample.
Data collection	We did not collect any data directly and this did not use any specific method, device, or instrument. We downloaded publicly available data published by 1) Johns Hopkins University Center for Systems Science and Engineering (COVID related data), 2) IPUMS National Historical GIS portal (socio-economic data), 3) U.S. National Oceanic and Atmospheric Administration, NOAA (temperature data), 4) Facebook (social connectedness and movement range data), and 5) SafeGraph (movement data).<br><br>These datasets were preprocessed in Python programming language and using open source packages (GeoPandas, Pandas, Numpy). The code that we used for data cleaning and preprocessing (along with the code for implementing STXGB model) is publicly available at https://github.com/geohai/COVID19-STGXB
Timing	From March29, 2020 to February 20, 2021
Data exclusions	No data (within the coterminous US) were excluded from the analyses.
Non-participation	This study did not use human participants. No participants dropped out/declined participation.
Randomization	This study did not use human participants. No participant was allocated into experimental groups.

Reporting for specific materials, systems and methods

We require information from authors about some types of materials, experimental systems and methods used in many studies. Here, indicate whether each material, system or method listed is relevant to your study. If you are not sure if a list item applies to your research, read the appropriate section before selecting a response.

Materials & experimental systems

n/a	Involved in the study
☒	☐ Antibodies
☒	☐ Eukaryotic cell lines
☒	☐ Palaeontology and archaeology
☒	☐ Animals and other organisms
☒	☐ Human research participants
☒	☐ Clinical data
☒	☐ Dual use research of concern

Methods

n/a	Involved in the study
☒	☐ ChIP-seq
☒	☐ Flow cytometry
☒	☐ MRI-based neuroimaging
